# The role of m^6^A modification in physiology and disease

**DOI:** 10.1038/s41419-020-03143-z

**Published:** 2020-11-08

**Authors:** Chuan Yang, Yiyang Hu, Bo Zhou, Yulu Bao, Zhibin Li, Chunli Gong, Huan Yang, Sumin Wang, Yufeng Xiao

**Affiliations:** Department of Gastroenterology, Xinqiao Hospital, Third Military Medical University, 400037 Chongqing, China

**Keywords:** Cancer, Endocrine system and metabolic diseases

## Abstract

Similar to DNA epigenetic modifications, multiple reversible chemical modifications on RNAs have been uncovered in a new layer of epigenetic modification. N6-methyladenosine (m^6^A), a modification that occurs in ~30% transcripts, is dynamically regulated by writer complex (methylase) and eraser (RNA demethylase) proteins, and is recognized by reader (m^6^A-binding) proteins. The effects of m^6^A modification are reflected in the functional modulation of mRNA splicing, export, localization, translation, and stability by regulating RNA structure and interactions between RNA and RNA-binding proteins. This modulation is involved in a variety of physiological behaviors, including neurodevelopment, immunoregulation, and cellular differentiation. The disruption of m^6^A modulations impairs gene expression and cellular function and ultimately leads to diseases such as cancer, psychiatric disorders, and metabolic disease. This review focuses on the mechanisms and functions of m^6^A modification in a variety of physiological behaviors and diseases.

## Facts

- A new research field: m^6^A has been known since the 1970s and serves as a new layer of epigenetic modification with the discovery of demethylases in recent years. m^6^A plays a broad and crucial role in almost all aspects of RNA metabolism.
- m^6^A exerts important roles in physiological regulation and its disruption may impair gene expression and cellular function and is involved in many diseases such as cancer, psychiatric disorders, and metabolic disease.
- Many enzymes concerning m^6^A may have not yet been identified. It is unclear how m^6^A changes the secondary structure of RNA and promotes the binding of RNA to proteins. Even the functions of the known m^6^A-related enzymes are not known.

## Open questions

- Are other enzymes related to m^6^A still to be discovered?
- How can m^6^A change the secondary structure of RNA and promote the binding of RNA to proteins?
- Can m^6^A be used as a biomarker for the early screening, diagnosis and treatment of cancer?
- Is m^6^A involved in a variety of pathways, and how can drugs that target specific m^6^A sites be developed to reduce unwanted side effects?

## Introduction

Epigenetics is one of the most intensely studied research fields, encompassing modifications that include DNA methylation, histone, and chromatin modifications. It is well known that in the central dogma of molecular biology, genetic information is transferred from DNA to RNA and then to proteins. Since there are reversible chemical modifications on DNA that can control the expression of genes, researchers suspect that similar modifications to RNA could be functional mediators of gene expression. In fact, multiple chemical modifications, such as m^6^A, 5-methylcytosine, and pseudouridine have been detected in a large subset of eukaryotic mRNAs. m^6^A is one of the most deeply researched modifications and plays a broad and crucial role in almost all aspects of physiological behavior. In the 1970s, RNA was found to possess complex base-methyl nucleoside patterns, and the distribution of these patterns in mRNA consists predominantly of m^6^A^1^. By treating HeLa cells with radioactive [methyl-^3^H] methionine, researchers found about one-third of the radioactivity was present in m^6^A in HeLa cell mRNA^2^. After the same treatment, approximately three-quarters of [^3^H] methyl label was in m^6^A in cytoplasmic simian-virus-40-specific RNA^3^. Most m^6^A residues are enriched at specific transcript landmarks, especially at the start of the last exon, in the 3′ UTRs and near stop codons^4,5^.

In the past few decades, the biological significance of m^6^A remained elusive. Only in recent years have several groundbreaking studies suggested that m^6^A plays a vital role in various aspects of RNA metabolism, including splicing, export, localization, translation, and stability^6–10^.These results promoted research into the biological significance of m^6^A. Lineage restriction of the yeast Saccharomyces cerevisiae during nutrient limitation depends on the mRNA methyltransferase activity of Ime4, suggesting a broad role of m^6^A in cell fate^11^. Spenito, a novel bona fide subunit of the methyltransferase complex, modulates neuronal functions and sex determination in Drosophila, suggesting a crucial role for m^6^A during the development of complex organisms^12^.

The methods used to detect m^6^A sites have been classified according to different purposes. LC-MS/MS is bases on liquid mass spectrometry with tandem mass spectrometry and is used to detect the overall m^6^A level on mRNA, revealed as molecular ion peaks and fragment ion peaks, and is a tool for performing qualitative and quantitative analysis of bases simultaneously^13^. The colorimetric method is similar to that of LC-MS/MS, but its procedure is simpler. Researchers can extract total RNA or enrich mRNAs with oligodT magnetic beads. m^6^A is then detected using specific capture and detection antibodies^14^. MiCLIP-seq and MeRIP-seq are high-throughput sequencing methods, but the former maps m^6^A sites by using anti-m^6^A antibodies and UV cross-linking techniques^15^, and it can be used to identify m^6^A sites at single-base resolution, in contrast to the latter. For MeRIP-seq, anti-m^6^A antibodies are incubated with RNA fragments to precipitate them for sequenceing^16^. m^6^A-IP-qPCR is used to quantify enriched RNA directly^17^, while dot blotting is used to detect the overall m^6^A methylation level in a more rapid and inexpensive way^18^. Additionally, there are some drawbacks to these different methods. For example, some MeRIP-seq data do not conform with validated data^19^.

m^6^A controls cell fate transition in mammalian embryonic stem cells (ESCs), and its disruption impairs ESC exit from self-renewal and ESC differentiation into several lineages^20^. These findings revealed that m^6^A is involved in a variety of physiological behaviors, and its dysfunction may be involved in the mechanisms associated with various diseases. In this review, we discuss the functions and biological consequences of m^6^A methylation, including physiology and disease, and the prospects for using m^6^A methylation as a new diagnostic biomarker and potential therapeutic target in disease.

## m^6^A writers, erasers, and readers

### Methyltransferases/writers

Researchers first identified a nucleic acid methyltransferase complex that comprises three components and is separable under nondenaturing conditions. The complex recognizes a highly conserved consensus site and specifically methylates only the N6 amino group of adenosine^21^. The stable heterodimeric complex of METTL3-METTL14 is the core of the methyltransferase complex and functions in cellular m^6^A deposition on mammalian nuclear RNAs^22^. Both the METTL3 and METTL14 proteins contain methyltransferase domains, but METTL3 is the catalytically active subunit that transfers a methyl group to RNA, and METTL14 plays a structural and noncatalytic role in substrate recognition, maintaining complex integrity and substrate RNA binding. METTL3 associates with chromatin and localizes to the transcriptional start sites (TSSs) of active genes, suggesting an independent role in transcription^23^. METTL3 alters the expression of splicing regulators, leading to unexpected splicing events, including insufficient DNA repair^24^. In addition, METTL3 promotes translation, and its depletion reduces m^6^A levels on the mRNA of the histone methyltransferase Ezh2, downregulating its expression at the translational level^25^. However, researchers have recently found that METTL3 can directly enhance the translation of certain mRNAs by recruiting eIF3 to the translation initiation complex independently of its methyltransferase activity and of downstream m^6^A reader proteins^26^.

The other subunit of the methyltransferase is Wilms’ tumor 1-associating protein (WTAP), a mammalian splicing factor that can interact with the METTL3-METTL14 complex and is essential for its localization to nuclear speckles and for its catalytic activity. The loss of WTAP reduces the RNA-binding capability of METTL3, suggesting that WTAP may promote the recruitment of the m^6^A methyltransferase complex to mRNA targets^27,28^. Further studies have demonstrated that KIAA1429 and RBM15 are also required for methylation^29,30^. The latter has been suggested to function in m^6^A modification in the long noncoding RNA X-inactive specific transcript (XIST) and in cellular mRNAs by binding and recruiting the m^6^A-methylation complex to specific sites in RNA.

Recently, researchers have found that METTL16, an active m^6^A methyltransferase, binds to the U6 snRNA and other ncRNAs, as well as numerous lncRNAs and pre-mRNAs, and is responsible for the m^6^A modification of A43 of the U6 snRNA, which base pairs with 50 pre-mRNA splice sites during splicing, expanding the mechanisms by which m^6^A is deposited on RNAs^31,32^. METTL16 was presumed to be a rRNA methyltransferase, as the ybiN gene in *E.coli* encodes the N^6^ position of A1618 in 23S rRNA specifically^33^. Interestingly, METTL16 also interacts with MALAT1 (metastasis-associated lung adenocarcinoma transcript 1) ENE+A (element for nuclear expression with a downstream A-rich tract)^34^. METTL16 regulates the expression of S-adenosylmethionine (SAM) synthetase MAT2A transcripts to modulate SAM homeostasis, which facilitates mouse embryonic development^35,36^. METTL5 was defined as the methyltransferase of 18S rRNA, ZCCHC4 was defined as the methyltransferase of 28S rRNA, and TRMT112 acts as a methyltransferase activator to stabilize METTL5 in cells^37^. METTL5 was also reported to be involved in the pluripotency and differentiation potential of mouse embryonic stem cells and the fly behavior of Drosophila^38,39^.

### Demethylases/erasers

The discovery of demethylases suggests that m^6^A modification is dynamic and reversible. Fat mass and obesity-associated protein (FTO), which was first found to be linked to obesity in population studies, partially colocalizes with nuclear speckles and exhibits efficient oxidative demethylation of abundant m^6^A in RNA^40,41^. Further studies have demonstrated that FTO is involved in the formation of two additional modifications derived from the prevalent m^6^A in mRNA, N6-hydroxymethyladenosine (hm^6^A), and N6-formyladenosine (f6A), which may regulate gene expression by affecting RNA-protein interactions^7^. Additionally, FTO regulates poly(A) sites and 3′ UTR length, and knocking it out results in substantial changes in pre-mRNA splicing, with a prevalence of exon skipping events^42^. In addition, the debate over m^6^Am continues. According to one report, m^6^A but not m^6^Am plays an important role in leukemia^43^, and the function of FTO is related to the location of m^6^A. In the nucleus, FTO is critical for m^6^A and m^6^Am in snRNAs, while in the cytoplasm, FTO is critical for cap m^6^Am in poly-A RNA^44^. Recently, FTO was shown to demethylate m^6^Am during snRNA biogenesis, which provides new insight into the classification of snRNA^45^. It is difficult to say whether FTO plays an oncogenic role in leukemia or is related to snRNA. ALKB homolog 5 (ALKBH5), another demethylase, oxidatively reverses m^6^A to affect mRNA export, metabolism, and the assembly of mRNA processing factors in nuclear speckles^46^. Unlike the mechanism by which methylases recognize their target transcripts via conserved consensus recognition sites, m^6^A, as a conformational marker, induces different conformational outcomes in RNAs depending on the sequence context, which promotes substrate recognition by the demethylases FTO and ALKBH5^47^.

### Readers

With methyltransferase and demethylases identified as the writers and erasers of m^6^A on mRNA, researchers focused on the readers of the m^6^A modification. The YT521-B homology (YTH) domain family has five members: YTHDF1, YTHDF2, YTHDF3, YTHDC1, and YTHDC2. All of these members can bind to m^6^A-modified RNA at the RRm^6^ACH consensus sequence through a conserved m^6^A-binding domain. The human YTH domain family 2 (YTHDF2) protein can selectively recognize and bind m^6^A-containing mRNA through a conserved core motif, G(m^6^A)C, to promote mRNA degradation. Its C-terminal domain is responsible for binding to m^6^A-modified mRNA, and its N-terminal domain promotes the localization of the YTHDF2-mRNA complex to cellular RNA decay sites^48^. Another m^6^A reader protein YTHDF1 actively interacts with translation machinery, increases translation efficiency and ultimately promotes protein synthesis, which enables fast changes in gene expression and controllable protein production^9^. Several studies show that YTHDF3 promotes protein synthesis in synergy with YTHDF1 and regulates YTHDF2-mediated methylated mRNA decay^10^. These three YTHDF proteins suggest a dynamic and multidimensional mechanism by which m^6^A regulates mRNA degradation and translation.

The nuclear m^6^A reader YTHDC1 can recruit and promote the interaction of pre-mRNA splicing factors with target mRNAs. For example, YTHDC1 recruits the pre-mRNA splicing factor SRSF3 (SRp20) and blocks SRSF10 (SRp38) mRNA binding, promoting exon inclusion in target mRNAs^49^. In addition, YTHDC1 interacts with SRSF3 and facilitates target RNA binding to both SRSF3 and NXF1, which promotes the export of methylated mRNA from the nucleus to the cytoplasm^50^.

**Fig. 1:**
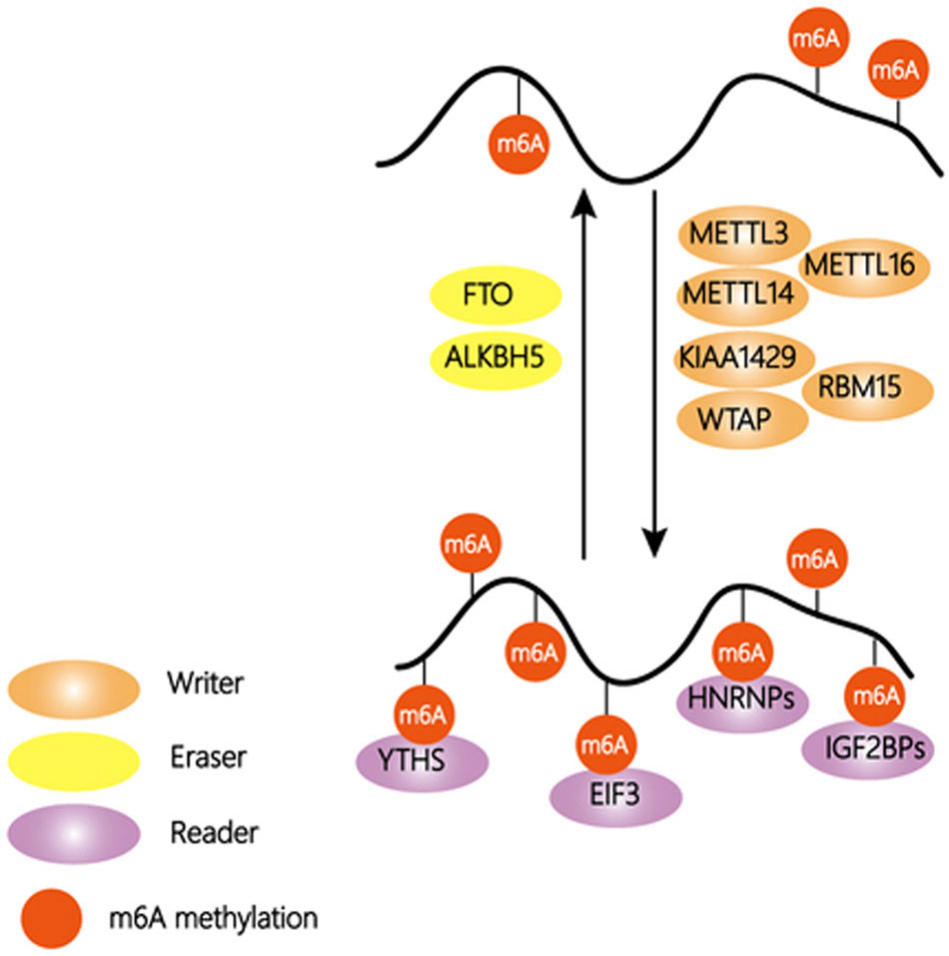
The regulation of m6A modification. M^6^A is added, removed and recognized by its writers, erasers and readers. METTL3-METTL14 is the core of the methyltransferase and functions in cellular m^6^A deposition on nuclear RNAs. WTAP is a subunit of the methyltransferase, which promotes the recruitment of the m6A methyltransferase complex to mRNA targets. KIAA1429 and RBM15 (RNA binding motif protein 15) are also required for the above process. FTO and ALKBH5 are two enzymes capable of removing m^6^A, exhibiting efficient oxidative demethylation activity of abundant m^6^A in RNA. YTH domain family, HNRNP protein family, IGF2BPs and eIF3 bind to m^6^A-modified RNA through conserved m6A-binding domains and play different roles in RNA metabolism.

The heterogeneous nuclear ribonucleoprotein (HNRNP) family and insulin-like growth factor 2 mRNA-binding proteins (IGF2BPs, including IGF2BP1/2/3) also bind m^6^A-bearing RNAs and serve as m^6^A readers. HNRNPA2B1 directly binds a set of nuclear m^6^A-methylated transcripts and regulates their alternative splicing in a similar manner as METTL3^51^. m^6^A can alter the local structure of mRNAs to promote the binding of transcripts to HNRNPG and HNRNPC, which affects the abundance and alternative splicing of target mRNAs^52,53^. In contrast to the mRNA-decay-promoting function of YTHDF2, IGF2BPs, a distinct family of m^6^A readers, bind mRNA transcripts through the consensus sequence GG(m^6^A)C and promote the stability and storage of their target mRNA^54^. In addition, eukaryotic initiation factor 3 (eIF3) directly binds 5′ UTR m^6^A to initiate translation without the cap-binding factor eIF4E, which suggests eIF3 is an m^6^A reader^55^. Recent studies have found that FMR1 and LRPPRC can also read m^6^A modifications, serving as m^6^A readers^56^. The mechanism is included in Fig. 1.

## m^6^A and normal physiological behaviors

### m^6^A and neurophysiology

#### Neurodevelopment

All known m^6^A enzymes and readers have been found in major brain cell types including neurons and neuroglia and their subtypes. m^6^A is extensively involved in neurodevelopment regulation which involves a complex system with multiple mechanisms. Studies have shown that m^6^A is important in the temporal control of mammalian cortical neurogenesis, promoting neurogenesis and neuronal development. Mettl14 knockout in embryonic mouse brains results in a prolonged radial glial cell (RGC) cycle, extending cortical neurogenesis into postnatal stages. METTL3 depletion in adult neural stem cells (aNSCs) not only inhibits the proliferation of aNSCs but also inhibits neuronal development and the morphological maturation of newborn neurons. Mechanistically, m^6^A depletion affects the decay of a set of transcripts related to transcription factors, neurogenesis, the cell cycle, and neuronal differentiation^57^. Meanwhile, m^6^A depletion on the mRNA of the histone methyltransferase Ezh2 downregulates its protein expression and consequent H3K27me3 levels, ultimately causes defects of neuronal development^25^. This modification of the Btg2 transcription factor also increases the efficiency of induced neuronal cell generation^58^.

m^6^A also regulates axon guidance via the translational control of the axon guidance receptor Robo3.1, which plays a key role in the midline crossing of spinal commissural axons. Mechanistically, YTHDF1 binds to endogenous m^6^A-modified Robo3.1 mRNA, upregulates its translation without affecting mRNA levels, and ultimately controls the guidance of pre-crossing commissural axons in the embryonic spinal cord^59^. A new m^6^A reader, proline rich coiled-coil 2A (Prrc2a), plays a crucial role in oligodendrocyte progenitor cell (OPC) proliferation and oligodendrocyte fate determination. Prrc2a can bind Olig2, a critical gene in oligodendrocyte development, and stabilize its mRNA. These results reveal Prrc2a as a novel m^6^A reader and provide a new pathway for therapies for hypomyelination-related neurological diseases^60^. Additionally, researchers found a new m^6^A reader fragile X mental retardation protein (FMRP), which functions during neural progenitor differentiation. FMRP preferentially binds m^6^A-modified mRNAs related to the regulation of neural differentiation to facilitate their nuclear export by the nuclear export protein CRM1, and its loss results in delayed neural progenitor cell cycle progression^61^.

The type and amount of m^6^A enzymes and binding proteins may exhibit distinct regional and subcellular distribution patterns in specific cell types. The complex neurodevelopment regulation system is likely to be based on this distribution, because of the long-distance distribution of mRNAs and proteins across axons and dendrites and the high cellular compartmentalization of the neuron (For a summary see Table 1).

**Table 1:** The role and mechanism of m^6^A in physiological behaviors.

Molecule	Molecular functions	Localization	Physiological functions and mechanism
*Normal physiological behaviors*			
METTL3/METTL14	mRNA decay	Cortical neural stem cells	Controls mammalian cortical neurogenesis by promoting decay of a set of transcripts related to transcription factors, neurogenesis, cell cycle, and neuronal differentiation^57^.
	Translation inhibition	Neural stem cells (NSCs)	Regulates neurogenesis and neuronal development by regulating Ezh2 expression at the translational level and ultimately affect H3K27me3 expression^25^.
METTL3	mRNA stabilization and Pre-mRNA splicing	Newborn cerebellar granule cells (CGCs)	Controls cerebellar development by regulating related RNA half-lives and splicing events in CGCs^131^.
YTHDF1	Translation activation	Spinal commissural neurons	Controls pre-crossing axon guidance in spinal cord by positively regulating translation of m6A-modified Robo3.1 mRNA^59^.
Prrc2a	mRNA stabilization	Oligodendrocyte progenitor cells	Controls oligodendroglia specification and myelination by stabilizing Olig2 mRNA through binding to a consensus GGACU motif in the Olig2 CDS^60^.
FMRP	mRNA nuclear export	Neural progenitors	Modulates neural differentiation through m^6^ A-dependent mRNA nuclear export^61^.
FTO	Not determined	Midbrain and striatum	Regulates activity of the dopaminergic midbrain circuitry by promoting demethylation of specific mRNAs related to DA transmission and controlling their proteins expression^62,63^.
YTHDF1	Translation activation	Hippocampus neurons	Facilitates learning and memory in response to neuronal stimuli by promoting translation of targeted transcripts^66^.
*Immunoregulation*			
METTL3	mRNA decay	Naive T cells and regulatory T cells	Controls T cell homeostasis by targeting the IL-7/STAT5/SOCS pathways^67,132^.
	Translation activation	Dendritic cell	Promotes dendritic cell activation and DC-based T cell response by increasing translation of certain immune transcripts^133^.
*Inflammatory response*			
METTL3	Pre-mRNA splicing	Human dental pulp cells (HDPCs)	Inhibits the LPS-induced inflammatory response of HDPCs by regulating alternative splicing of MyD88^134^.
*Stem cell fate*			
METTL3	mRNA decay	Mouse embryonic stem cells	Promotes resolution of murine naive pluripotency toward differentiation by reducing the stability of key naïve pluripotency-promoting transcripts, including core pluripotency regulators Nanog^20,69,70^.
METTL3/METTL14	mRNA decay	Mouse embryonic stem cells	Maintains self-renewal capability by destabilizing developmental regulators^135^.
METTL3	Not determined	Mouse embryonic fibroblasts	Promotes the reprogramming of mouse embryonic fibroblasts to pluripotent stem cells^136^.
YTHDF2	mRNA decay	Hematopoietic stem cells	Inhibits hematopoietic stem cells self-renewal by promoting decay of transcripts which encodes transcription factors necessary for stem cell self-renewal^137,138^.
	mRNA decay	Arterial endothelial cells	Promotes EHT and the generation of the earliest haematopoietic stem/progenitor cells (HSPCs) through mRNA decay of the arterial endothelial genes notch1a and rhoca^139^.
*Gametogenesis*			
METTL3/METTL14	Alternative splicing and translation activation	Spermatogonium	Controls spermatogonial differentiation and meiosis initiation by regulating alternative splicing and translation of haploid-specific genes that are essential for spermiogenesis^140,141^.
ALKBH5	Pre-mRNA splicing	Spermatocytes and round spermatids	Controls splicing and stability of long 3′-UTR mRNAs in male germ cells^142^.
FTO	mRNA decay	Hela cells	Inhibited by MA2 is accompanied with elevated m6A levels in cyclin-dependent kinases, accelerating their destabilization and impairing the cell cycle progression^143^.
YTHDC2	mRNA decay	Spermatogonia	Facilitates a clean switch from mitosis to meiosis in mouse germ cells by downregulating genes expression that are important for mitosis^144–147^.
YTHDC1	Alternative polyadenylation and Pre-mRNA splicing	Mouse oocyte	Controls mouse oocyte development by regulating alternative polyadenylation and splicing^148^.
YTHDF2	Regulate transcript dosage	Oocyte	Post-transcriptionally regulates transcript dosage during oocyte maturation, crucial for oocyte growth and maturation^149^.

#### Learning and memory

Studies have demonstrated that m^6^A is dynamically upregulated in the mouse medial prefrontal cortex (mPFC) in response to behavioral training, suggesting a close association with behavioral adaptation. The mechanism likely involves m^6^A increasing the targeting of plasticity-related genes and promoting their efficient translation and rapid degradation. Therefore, FTO knockdown in the mPFC promotes the consolidation of cued fear memory^62,63^. m^6^A also plays a significant role in reward learning by regulating related protein expression in the DA signaling pathway. FTO depletion impairs D2/3R (dopamine receptor type 2 and type 3) signaling in the midbrain of mice through the disruption of demethylation on specific mRNAs related to DA transmission. and related neuronal activity and behavioral responses^64^. In addition, FTO variants modulate the connectivity in a basic reward circuit of the neostriata-prefrontal regions, showing that genetic predisposition can also affect other disorders with altered D2R-dependent impulse control, such as addiction^65^. FTO is strongly associated with learning, memory, and behavioral training. However, the multiple underlying neurobiological mechanisms by which FTO influences the brain and behavior through m^6^A remains unknown,

Recent studies also show that YTHDF1 promotes learning and memory in response to neuronal stimuli by facilitating the translation of targeted transcripts in the adult mouse hippocampus. Its deletion impairs hippocampal synaptic transmission and long-term potentiation, causing learning and memory defects^66^.

### m^6^A and immunoregulation

#### m^6^A and T-cell homeostasis

Recent studies have demonstrated that m^6^A plays a crucial role in controlling T-cell homeostasis. m^6^A modification in IL-7-induced naïve T cells promotes the degradation of the suppressor of cytokine signaling (SOCS) genes, which are involved in inhibiting JAK-STAT signaling. IL-7-STAT5 signaling is activated and initiates naïve T cell reprogramming for proliferation and differentiation^67^.

#### Inflammatory response

Dental pulp inflammation is a typical inflammatory disease that is characterized by the partial accumulation of inflammatory mediators. Researchers identified lnc-Dpf3, which suppresses CCR7-mediated DC migration. Mechanistically, CCR7 stimulation decreases the m^6^A levels of lnc-Dpf3, relieves m^6^A-dependent degradation, and ultimately upregulates its expression. lnc-Dpf3 feedback suppresses the HIF-1a-dependent transcription of the glycolytic gene Ldha by directly binding to HIF-1a, subsequently inhibiting DC glycolytic metabolism and migratory capacity^68^.

### m^6^A and stem cell fate

ESCs are cells isolated from the early embryo or original gonad that have two main features: self-renewal and multidirectional differentiation potential. Recent studies have found m^6^A promotes the resolution of murine naïve pluripotency toward differentiation by reducing the stability of key naïve pluripotency-promoting transcripts, including the core pluripotency regulator Nanog^20,69,70^. The results reflected the effect of m^6^A on the differentiation of ESCs, however, many studies show conflicting results. Zc3h13-WTAP-Virilizer-Hakai is an evolutionarily conserved complex that contributes to the regulation of RNA m^6^A methylation. ZC3H13, a zinc-finger protein, maintains mouse embryonic stem cell (mESC) self-renewal by anchoring the above complex in the nucleus and and facilitating m^6^A methylation. Zc3h13 depletion impairs self-renewal and triggers mESC differentiation^71^. The vast majority of large intergenic noncoding RNAs (lincRNAs) are necessary for the maintenance of ESC pluripotency^72^. The role of m^6^A in stem cell fate determination has also been confirmed in hematopoietic stem cells (HSCs).

The interaction of m^6^A with stem cell fate has been found in NSCs, glioblastoma (GBM), and leukemia, and the relevant mechanisms will be described in the following sections.

m^6^A plays conflicting and dual roles in stem cell fate, especially ESCs. Many studies have found that m^6^A reduces pluripotency and promotes differentiation, whereas others have shown the opposite. This dual role is likely due to the widespread presence of m^6^A in pluripotency regulators and developmental regulators. Meanwhile, abundant RNA-binding proteins including m^6^A reader proteins and non-reader proteins directly or indirectly interacts with m^6^A-modified RNA, forming a complex network that regulates stem cell fate. This network allows stem cells to choose to self-renew or differentiate at the right time, which would make m^6^A play a different or even opposite role. The existence of above readers such as eIF3, LRPPRC, FMR1, and Prrc2a are also likely involved in regulating stem cell fate.

### m^6^A and gametogenesis

By analyzing the m^6^A mRNA methylomes of mouse spermatogenic cells at five different developmental stages, researchers found dynamic changes in m^6^A abundance during different developmental stages of spermatogenesis, suggesting crucial role of m^6^A in spermatogenesis.

Recent studies indicate the enrichment of m^6^A RNA modification in most key regulators of spermatogonial stem cell progenitor cells, including Plzf, Id4, Dnmt3b, and Sohlh2. m^6^A could provide a marker to these transcripts to modulate their coordinated translation (For a summary see Table 1).

## m^6^A and disease

### m^6^A and cancer

#### m^6^A and cancer stem cell pluripotency

Cancer stem cells (CSCs) constitute a rare subclass of neoplastic cells within tumors that have a stem cell-like capacity to self-renew and undergo multidirectional differentiation. We have described the role of m^6^A in determining the fate of stem cells above, and discuss the role of m^6^A in cancer stem cells as a new research direction here.

The abnormal or blocked differentiation of HSCs is an important feature of myeloid hematological malignancies. Because HSCs have well-defined cell differentiation trajectories and serve as ideal model systems, we chose to focus on HSCs to discuss the relationship between m^6^A and cancer stem cell pluripotency. Acute myeloid leukemia (AML) is a cancer of haematopoietic progenitor cells characterized by the proliferation of blast cells and loss of normal haematopoiesis^73^. Many studies have shown that METTL3 is highly expressed and that the m^6^A levels of transcripts are increased, including myelocytomatosis (MYC), B cell lymphoma 2 (BCL2), and phosphatase and tensin homolog (PTEN) transcripts, in human AML. Upregulated m^6^A levels promote the translation of these mRNAs and thereby retain pluripotency properties and inhibit cell differentiation^23,74^. Similar to METTL3, METTL14 targets MYB and MYC mRNAs and increases their expression, suggesting crucial roles in myelopoiesis and leukemogenesis^75^. Interestingly, the FTO-mediated decrease in m^6^A may play oncogenic roles in certain types of AML. A suppressor of cytokine signaling box-2 (ASB2) and retinoic acid receptor alpha (RARA), which promote normal hematopoiesis and the ATRA-induced APL differentiation of leukemia cells, are downregulated by FTO in an m^6^A-dependent manner^76^. Related to the role of FTO, R-2-hydroxyglutarate (R-2HG) increases the m^6^A levels of MYC/CEBPA mRNAs through inhibition of FTO, reduces their stability and downregulates their expression, exhibiting anti-proliferation effects in leukemia^43^. Through the analysis of these results, we found that m^6^A plays a dual function in AML cell pluripotency by regulating the expression of key genes. This regulatory network is complex. Widespread m^6^A does drive cells in a specific direction, it is a part of a comprehensive regulation system, and the final result is determined by various factors.

Intratumoral hypoxia is a critical factor that drives breast cancer progression. ZNF217, an m^6^A methyltransferase inhibitor targeting METTL3, is upregulated in hypoxia-induced breast cancer cells. ZNF217 increases pluripotency factor KLF4 and NANOG expression in an m^6^A-dependent manner, promoting pluripotency factor expression and breast cancer stem cell (BCSC) specification^77^. Glioblastoma stem-like cells (GSCs) are a class of self-renewing cells related to GBM origin, growth, invasion, and recurrence. Studies found that METTL3 overexpression suppresses GSC proliferation and self-renewal by increasing m^6^A levels and decreasing the expression of ADAM19, which has critical biological functions in GSCs^78^. Other studies demonstrated that ALKBH5 is highly expressed in GBM and promotes GSC tumorigenicity. ALKBH5 decreases m^6^A on FOXM1 nascent transcript and increases the expression of FOXM1, which plays a pivotal role in GSC proliferation and self-renewal, thereby increasing its expression^79^. CSCs represent a reservoir of self-sustaining cells that cause many types of cancers. The role of m^6^A in CSCs is complex and interesting, and relevant studies are not described here. We believe that the role of m^6^A in CSC is a critical cancer research direction.

#### m^6^A is involved in cancer cell migration and tumor metastasis

The studies that show m^6^A regulating cancer cell migration and metastasis have been an important and full-fledged aspects of cancer research. Here, we discuss many studies demonstrating the possibility of inhibiting tumor migration through the regulation of m^6^A.

Hepatocellular carcinoma (HCC) is the major type of primary hepatic carcinoma. METTL3 is prominently upregulated and promotes the migration of HCC cells by targeting suppressor of cytokine signaling 2 (SOCS2) causing its degradation. Downregulated SOCS2 cannot effectively serve as a tumor suppressor in HCC^80^. However, as another m^6^A writer, METTL14 shows decreased expression in tissues and advanced metastasis capability in HCC. METTL14 increases the m^6^A abundance of pri-miR126 and then promotes its interaction with DGCR8, positively modulating the pri-miR-126 process which has been found to suppress metastasis^76^.

In pancreatic cancer, ALKBH5 is weakly expressed and inhibits cell migration and invasion by demethylating the lncRNA KCNK15-AS1 and regulating KCNK15-AS1-mediated cell motility^81^. Additionally, YTHDF2 has the dual effect of promoting the proliferation and inhibiting the migration and invasion of pancreatic cancer cells; this effect is called the “migration proliferation dichotomy”. YTHDF2 also regulates the epithelial–mesenchymal transition (EMT) via the downregulation of total yes-associated protein (YAP) mRNA which has been reported to be closely related to the EMT in pancreatic cancer cells. Previous studies found that YAP has two m^6^A sites in the CDS and exon region. It is reasonable to think that YTHDF2 might directly bind to YAP mRNA to decrease its stability^82^.

Many studies have been carried out on the function of m^6^A in cancer cell migration and tumor metastasis, but many precise mechanisms such as the dynamic regulation of the co-transcriptional installation of m^6^A RNA methylation remain unknown. Meanwhile, due to different target mRNA and reader, m^6^A act antipodal roles in HCC, suggesting a complex regulator control system that needs further research.

#### m^6^A regulates cancer cell proliferation

In addition to abnormal cell migration, uncontrolled proliferation is a main characteristic of tumor growth. m^6^A has been shown to regulate cancer cell proliferation in many kinds of cancers.

In breast cancer, METTL3 participates in a positive feedback loop comprising HBXIP/let-7g/METTL3/HBXIP that promotes proliferation. HBXIP, a type of oncoprotein associated with the aggressiveness of breast cancer, upregulates METTL3 by suppressing miRNA let-7g, and then METTL3 promotes the expression of HBXIP in an m^6^A-dependent manner^83^.

Researchers found that METTL14 mutations or METTL3 downregulation in endometrial cancer increases cell proliferation and tumorigenicity. Mechanistically, a low abundance of m^6^A downregulates the negative AKT regulator PHLPP2 and upregulates the positive AKT regulator mTORC2. The AKT signaling pathway is thus significantly activated and promotes the growth of endometrial cancer^17^.

In prostate cancer (PCa), YTHDF2 is frequently upregulated and promotes the proliferation of cancer cells. miR-493-3p is negatively correlated with YTHDF2 and suppresses tumor cell proliferation. YTHDF2 decreases m^6^A levels, and miR-493-3p increases m^6^A levels in PCa. These two crucial m^6^A regulators are involved in the progression of PCa by indirectly modulating m^6^A levels. In addition, miR-493-3p directly targets the 3′-UTR of YTHDF2 and reduces its expression^84^. These results preliminarily indicate a role for m^6^A in regulating PCa, but the specific mechanism remains to be studied.

#### m^6^A and chemoradiotherapy resisitance

FTO enhances the chemoradiotherapy resistance of cervical squamous cell carcinoma (CSCC) by positively regulating β-catenin expression via mRNA demethylation and in turn increasing excision repair cross-complementation group 1 (ERCC1) activity^85^. In GSCs, METTL3 is upregulated and confers radio resistance to GSCs. Mechanistically, METTL3 directly targets the 3′UTR of the SOX2 transcript, enhancing its stability. SOX2 has been shown to be associated with radiation resistance in various cancers. METTL3 causes radio resistance of GSCs through SOX2-dependent enhanced DNA repair^86^. There are few studies on m^6^A and chemoradiotherapy resistance, but research results may provide potential molecular targets for cancer therapy.

#### m^6^A and antitumor immunity

**Fig. 2:**
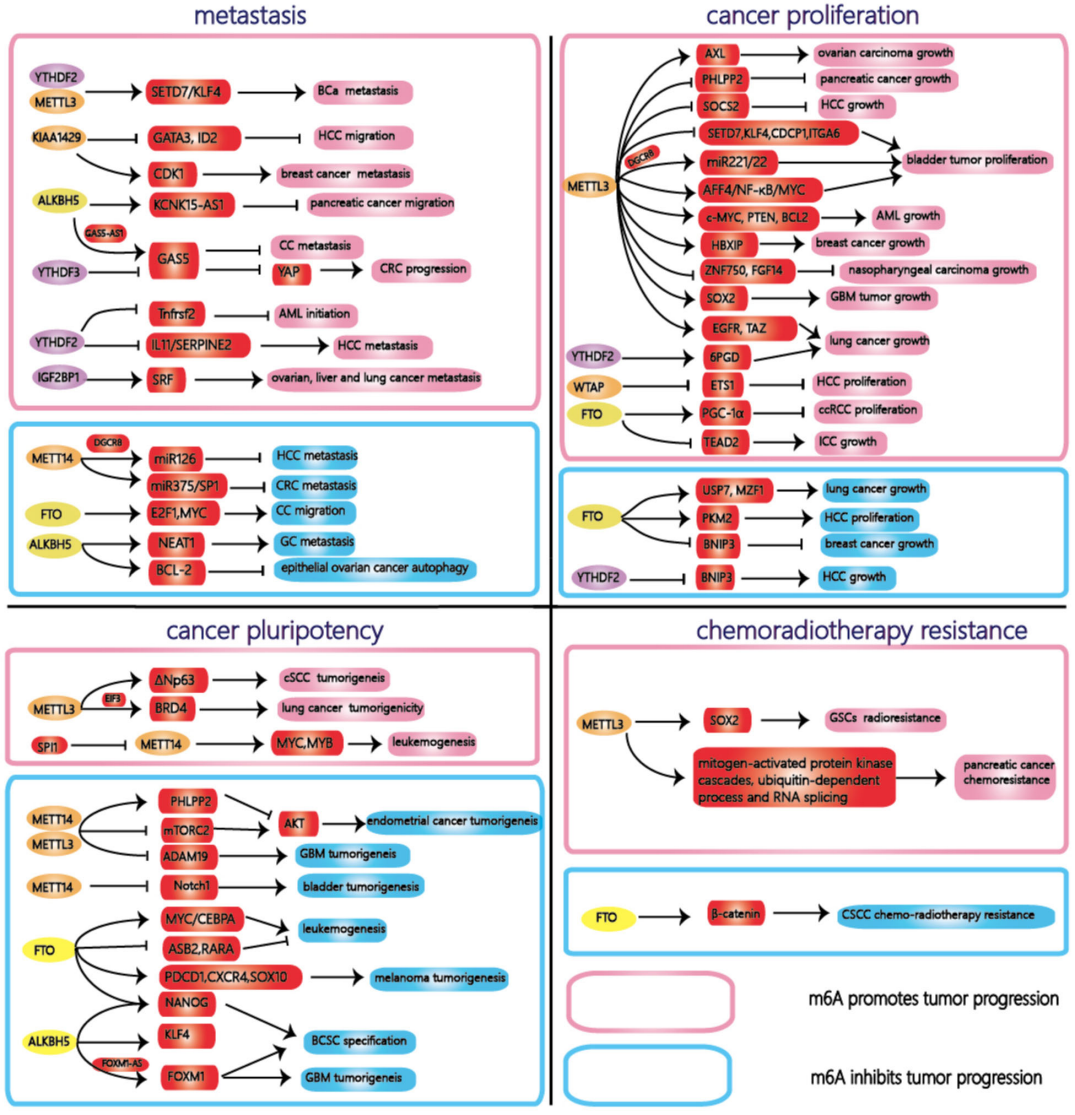
The role of m6A in different cancers. M^6^A plays diverse roles in different cancer, and even plays the opposite roles in a type of cancer. On the on hand, m^6^A promotes tumor progression by increasing oncogene expression and decreasing tumor suppressor gene expression. On the other hand, m^6^A suppresses tumor progression in opposite ways. Specific functions of m6A in the main text.

Recently, researchers demonstrated that m^6^A controls antitumor immunity via YTHDF1 in dendritic cells (DCs). The depletion of YTHDF1 in classical DCs can enhance the cross-presentation of tumor antigens and the cross-priming of CD8+T cells. Mechanistically, m^6^A-modified transcripts encoding lysosomal proteases are recognized and bound by YTHDF1, which promotes their translation in DCs and the subsequent suppression of the cross-presentation of DCs. These results reveal a previously unrecognized mechanism of immune evasion and show that YTHDF1 may be a potential therapeutic target for immunotherapy^87^. FTO also increases resistance to anti-PD-1 blockade immunotherapy by decreasing the number of m^6^A-modified mRNA transcripts, thus reducing their decay in melanoma^88^ (For a summary see Fig. 2).

### m^6^A and neuronal disorders

#### Nerve injury and malformation

Peripheral axon injury mostly increases m^6^A levels in the adult mouse dorsal root ganglion (DRG), with an enrichment of mRNA related to encoding regeneration-association genes (RAGs) and translational machinery, suggesting that m^6^A plays an essential role in the axonal regeneration of adult DRG neurons. Mettl14 and YTHDF1 are required for SNL-induced global protein synthesis and the robust axonal regeneration of DRG neurons; the depletion of these proteins reduces injury-induced protein translation in adult DRGs and negatively impacts functional axon regeneration in the peripheral nervous system in vivo. Mettl14 is also required in the central nervous system for the PTEN-deletion-induced robust axonal regeneration of adult retinal ganglion neurons. These results reveal that m^6^A methylation serves a crucial role in normal physiology and in responses to pathological stimuli in the adult mammalian nervous system^89^. METTL5 is abundant in the nucleus and synapses of hippocampal neurons, and its deletion leads to microcephaly in zebrafish^90^.

#### Psychiatric disorders

The FTO rs9939609 A variant may be connected with a lower risk of depression independently of its effect on BMI, and its effect on major depression (MDD) differ across MDD subtypes^91,92^. The ALKBH5 rs12936694 variant also showed an allelic association and a genotypic association with MDD^93^. Further studies have shown that the FTO SNP rs8050136 is involved in modulating the risk for attention-deficit/hyperactivity disorder (ADHD), especially in children who are not exposed to maternal smoking during pregnancy (MSDP). These results may provide a possible link between the physiopathology of ADHD and obesity^94^. Many transcripts modified by m^6^A are related to mental disorders such as autism and schizophrenia^95^. Elevated METTL3 and decreased FTO expression were associated with synaptic and neuron development in Alzheimer’s disease (AD)^96^. Arsenite induced elevated m^6^A modifications with deficiency of dopaminergic neurotransmission, and FTO participated in the process^97^. In Parkinson’s disease (PD), decreased m^6^A modification mediated by FTO overexpression led to *N*-methyl-d-aspartate (NMDA) receptor 1 expression, which promoted oxidative stress and induced dopaminergic neuron apoptosis^98^.

### m^6^A and osteoporosis

Bone-marrow-derived mesenchymal stem cells (BMSCs) have been demonstrated to differentiate into different cell lineages^99^. An imbalance in the differentiation of adipocytes and osteoblasts from BMSCs is an important factor leading to osteoporosis^100^. Recently, studies have revealed the potential involvement of m^6^A in bone homeostasis and osteoporosis. Growth differentiation factor 11 (GDF11) is a key factor in the development of osteoporosis^101^. Researchers have found that GDF11 controls the shift in osteoporotic MSC fate to adipocytes and inhibits bone formation during osteoporosis in an m^6^A-dependent manner. GDF11 upregulates FTO in a C/EBPα-dependent manner in osteoporotic BMSCs. FTO can reduce m^6^A levels in the mRNA of peroxisome proliferator-activated receptor gamma (Pparg) and subsequently promote its expression, which has been demonstrated to promote adipocyte differentiation from BMSCs^102^. These findings identify a novel axis for adipocyte and osteoblast differentiation, as well as osteoporosis. Unlike the role of GDF11, miR-149-3p has been suggested to inhibit the adipogenic differentiation of BMSCs and promote osteogenic differentiation and osteoblast extracellular matrix maturation and mineralization by targeting FTO^103^. METTL3 has also been found to regulate the fate of BMSCs and osteoporosis. Its overexpression prevents mice from developing estrogen-deficiency-induced osteoporosis, and its loss induces the pathological features of osteoporosis in mice. Mechanistically, METTL3 depletion reduces the translation efficiency of Pth1r (parathyroid hormone receptor-1), which regulates osteogenic and adipogenic responses in vivo and ultimately results in bone impairment and marrow fat accumulation^104,105^. These findings may provide many novel strategies for the treatment of osteoporosis. The role of m^6^A in osteoporosis is mainly reflected in the regulation of BMSCs, suggesting the importance of the complex and fine-tuned regulation of m^6^A on stem cell fate. These results also highlight the far-reaching implications of m^6^A in disease treatment.

### m^6^A and metabolic disease

#### Obesity and lipid metabolism

FTO was first found to be linked to obesity in multiple human populations and ethnic groups in population studies^41,106,107^. There is a common variant, rs9939609, in the first intron of the FTO gene that is associated with elevated body mass index (BMI) and leads to childhood and adult obesity^108^. Obesity is one of the main risk factors for the development of cardiovascular disease (CVD) and hypertension. A meta-analysis revealed that this FTO variant is significantly associated with the risk of CVD and hypertension^109,110^. Many studies have revealed that the polymorphisms in the first intron of FTO control the expression of RPGRIP1 similar to RPGRIP1L and iroquois-related homeobox 3 (IRX3). RPGRIP1L, a ciliary gene near the FTO locus, is related to diminished AcIII-positive cilia and the impaired assembly of the leptin receptor and is probably responsible for the obesity susceptibility signal at the FTO locus^111^. IRX3, whose expression is associated with obesity-associated SNPs, directly regulates body mass and composition with browning of white adipose tissue^112^. However, most effects caused by FTO are hard to distinguish from the function of IRX3. The status of FTO in obesity alone does not reveal an effect, especially when the level of IRX3 is changed through FTO knockdown or overexpression.

Results from loss-of-function studies and overexpression studies in mice have revealed that FTO plays an important role in controlling body weight and fat mass and functionally regulates energy homeostasis by controlling energy expenditure. The inactivation of the FTO gene results in postnatal growth retardation and reduced adipose tissue and lean body mass in mice^113^. A missense mutation (I367F) within the C-terminal domain of FTO leads to a reduction in fat mass and an increase in energy expenditure with unchanged physical activity^114^. By contrast, FTO overexpression increases food intake and results in a dose-dependent increase in the body and the fat mass of mice independently of their receiving a standard or a high-fat diet^115^.

Further studies have found that FTO inhibits the adipogenesis of pre-adipocytes by controlling cell cycle progression at the early stage of adipogenesis. Mechanistically, FTO depletion significantly upregulates the m^6^A levels of CCNA2 and CDK2 mRNA, causes them to be recognized and degraded by YTHDF2 and ultimately prolongs cell cycle progression to suppress adipogenesis^116^.

m^6^A mRNA methylation is involved in hepatic lipid metabolism. BMAL1 is a key component of the mammalian clock gene regulatory network associated with regulating metabolism^117^. FTO promotes the mitochondrial recruitment of STAT3 at the expense of its nuclear localization, affecting oxidative metabolism and the expression of leptin-targeted genes^118^. Further studies have found that FTO mRNA and protein levels were significantly increased in nonalcoholic fatty liver disease (NAFLD), which enhances lipogenesis and oxidative stress^119^.

#### Glucose metabolism

The association of genetic variations in FTO with the risk of type 2 diabetes in multiple human populations and ethnic groups reveals that FTO plays an important role in glucose metabolism^120–122^. Reported findings support the hypothesis that hepatic FTO is involved in the regulation of glucose homeostasis by inhibiting gluconeogenic gene expression in the liver under the of effects of glucose and insulin^123^ (For a summary see Table 2).

**Table 2:** The role and mechanism of m^6^A in human disease.

Molecule	Molecular functions	Localization	Disease and mechanism
*Neuronal disorders*			
METTL14/YTHDF1	Translation activation	DRG neurons	Facilitates axon regeneration of adult DRG neurons by promoting injury-induced protein synthesis^89^.
METTL5	Unknown	Hippocampal neurons	Its deletion causes microcephaly in zebrafish^90^.
FTO/ALKBH5	Unknown	Not sure	Major depression^91–93^.
FTO	Unknown	Not sure	Attention-deficit/hyperactivity disorder^94^.
FTO	Unknown	Dopaminergic cells	Decreased m6A modification led to NMDA receptor 1 expression, promoting oxidative stress, inducing dopaminergic neuron apoptosis^97,98^.
*Osteoporosis*			
FTO	mRNA stabilization bone	Bone mesenchymal stem cell	Promotes the shift of osteoporotic BMSC fate to adipocyte and inhibited bone formation during osteoporosis by increasing expression of Pparg^102^.
METTL3	Translation activation	Bone mesenchymal stem cell	Prevents the mice from estrogen deficiency-induced osteoporosis by increasing the translation efficiency of Pth1r^104,105^.
*Metabolic disease*			
FTO	Not determined	Human fibroblasts	The polymorphisms in the first intron of FTO controls expression of RPGRIP1 like (RPGRIP1L) which is related to diminished AcIII-positive cilia and impaired convening of the leptin receptor^111^.
	Pre-mRNA splicing	Pre-adipocytes	Regulates adipogenesis by controlling exonic splicing of adipogenic regulatory factor RUNX1T1^48^.
	mRNA stabilization	Pre-adipocytes	Regulates adipogenesis by controlling cell cycle progression in an m6 AYTHDF2-dependent manner^116^.
METTL3	mRNA decay	HepG2 cells	Regulates circadian clock of hepatic lipid metabolism by reducing stability of PPaRa mRNA^150^.
FTO	Transcription activation	Human hepatic HuH7 cells,	Regulates gluconeogenesis by promoting the expression of PCK1 and G6PC through the activation of and interaction with transcription factors such as STAT3 and C/EBP-β or upregulated ATF4^123,151–154^.
*Viral infection*			
ALKBH5	Nucleus retention	Macrophages	Demethylates those m6A-modified antiviral transcripts and enforces their retention in the nucleus to inhibits antiviral innate responses^155^.
METTL3/YTHDF2	mRNA decay	Human cytomegalovirus	Serves as negative regulators of interferon response by promoting interferon mRNAs decay and consequently facilitating viral propagation^125^.
METTL3/METTL14	mRNA nuclear export	CD4 T cells	Increases viral replication by promoting nuclear export of viral RNA through Rev-RRE interactions^156^.
METTL3/YTHDF2	Not determined	iSLK.219 cells and iSLK.BAC16 cell	Regulates expression of viral gene and production of virion by post-transcriptionally controlling ORF50 expression^157^.
	Translation activation	BSC40 cells	Enhances viral gene expression and replication by promoting the translation of viral late transcripts^127^.

### m^6^A and viral infection

The cytosolic RIG-I-like receptors (RLRs) play a crucial role in activating innate immune signaling by recognizing and binding to Invading pathogen nucleic acids. Recent studies have found that viral transcripts modified by m^6^A poorly bind to RIG-I and cannot effectively stimulate RIG-I-mediated antiviral signaling^124^. Interestingly, studies have revealed an opposite role for m^6^A in antiviral immunity in which m^6^A is required for the propagation of human cytomegalovirus (HCMV). METTL3/METTL14 and YTHDF2/YTHDC1 are upregulated in primary human foreskin fibroblasts infected by HCMV. METTL3 depletion decreases the m^6^A levels of IFNB mRNA, enhances its stability and sustains IFN-β production^125^. Further studies have also demonstrated that m^6^A-modifying enzymes regulate responses to nonmicrobial dsDNA in uninfected cells, which could shape host immunity and lead to autoimmune disease^126^.

Recently, researchers identified multiple m^6^A sites on prototypic polyomavirus simian virus 40 (SV40) mRNAs, which play a positive role in the regulation of SV40 gene expression. The inactivation of these m^6^A sites or endogenous YTHDF2/METTL3 inhibits SV40 replication in BSC40 cells. m^6^A sites present in the VP1 open reading frame (ORF) in the SV40 late region promote VP1 mRNA translation. The drug 3-deazaadenosine (DAA), a global inhibitor of methylation, can inhibit viral replication via the depletion of m^6^A^127^ (Summarized in Table 2).

## Concluding remarks and future perspectives

As increasing evidence suggests that m^6^A plays a crucial role in cancer, using m^6^A as a target for the early screening, diagnosis, and treatment of cancer seems to be feasible. For instance, R-2HG exhibits antitumor activity in AML and glioma by inhibiting FTO, but its roles in cancer are complex. For certain genes, m^6^A may promote the development of cancer, but for other genes, modification may serve as a suppressor of cancer. The modification occurs not only on eukaryotic mRNAs but also on noncoding RNAs. It is difficult to define a uniquely promoting or suppressing role of m^6^A in the development of human diseases. Among the enzymes participating in m^6^A modification, METTL3 and FTO seem to play more important roles in the progression of different diseases, and their function may serve as blueprints for translational research and therapeutics. Ubiquitination and SUMOylation have been reported to affect their demethylase or methyltransferase activity. Further studies are needed before m^6^A can be used in clinical therapies.

m^6^A is also involved in a variety of physiological behaviors such as neurodevelopment, T cell homeostasis, glucolipid metabolism and gametogenesis, and its disruption leads to various diseases, including addiction, autoimmune disease, metabolic disease, and infertility. For example, FTO is essential for neurodevelopment, as its depletion leads to the reduced proliferation and neuronal differentiation of NSCs, which ultimately reduces the number of NSCs in both the SGZ and SVZ regions. Mechanistically, the loss of FTO alters the m^6^A modification of key mRNAs and regulates their expression, especially affecting genes involved in the BDNF pathway^128^. In the last few years, there have been many breakthroughs in m^6^A, which has become an attractive target for therapy. For example, the drug 3-deazaadenosine (DAA) can inhibit viral replication via the depletion of m^6^A, but many queries of the mechanism remain. DAA acts as an inhibitor of S-adenosylhomocysteine hydrolase and it has anti-HIV activity. The reason of the inhibited level of m^6^A modification remains unclear. The expression of methyltransferases may be suppressed and that of demethylases may be promoted. However, the mechanism explaining theimbalance of m^6^A is also unclear. There may be many enzymes functionally related to m^6^A that have not yet been identified. It is unclear how m^6^A changes the secondary structure of RNA and promotes the binding of RNA to proteins. Even the functions of the known m^6^A-related enzymes are not known. Moreover, m^6^A is involved in a variety of pathways, and m^6^A-related drugs could likely cause unwanted side effects. Relevant studies on m^6^A are still focused on mechanisms and functions, and there is still a long way to go before clinical applications and drug development get processed.

Whole-transcriptome m^6^A sequencing of major human fetal tissues has been conducted, revealing a positive correlation between m^6^A level and gene expression homeostasis. The findings show the enrichment of m^6^A for genes with CpG-rich promoters, and these promoters and gene variations regulate m^6^A modification to drive human development and disease^129^. For co-transcription regulation, m^6^A mediation of YTHDC1 recruits KDM3B to m^6^A-associated chromatin regions, leading to H3K9me2 demethylation and enhanced gene expression^130^. Future research will focus on the genetic regulation of m^6^A and its role and mechanism in human health and diseases.
